# A novel role for β2-microglobulin: a precursor of antibacterial chemokine in respiratory epithelial cells

**DOI:** 10.1038/srep31035

**Published:** 2016-08-09

**Authors:** Shean-Jaw Chiou, Chan-Chi Wang, Yan-Shen Tseng, Yen-Jung Lee, Shih-Chieh Chen, Chi-Hsien Chou, Lea-Yea Chuang, Yi-Ren Hong, Chi-Yu Lu, Chien-Chih Chiu, Michel Chignard

**Affiliations:** 1 Department of Biochemistry, College of Medicine, Kaohsiung Medical University, Kaohsiung, Taiwan; 2 Center for Research Resources and Development, Kaohsiung Medical University, Kaohsiung, Taiwan; 3 Graduate Institute of Medicine, College of Medicine, Kaohsiung Medical University, Kaohsiung, Taiwan; 4 Research Center for Environmental Medicine, Kaohsiung Medical University, Kaohsiung, Taiwan; 5 Department of Biotechnology, Kaohsiung Medical University, Kaohsiung, Taiwan; 6 Unité de Défense Innée et Inflammation, Inserm U874, Institut Pasteur, Paris, France; 7 Centre de Recherche Saint-Antoine, UMR_S 938 - UPMC/Inserm, France

## Abstract

We analyzed a panel of cationic molecules secreted in the culture medium of human respiratory epithelial cells (REC) upon activation by IL-1β and different pathogen-associated molecular patterns. A 9 kDa fragment derived from β2-microglobulin (B2M) was identified and named shed 9 kDa B2M (sB2M-9). The primary structure of sB2M-9 was revealed to increase its pI value that potentially could play an important role in innate defense. sB2M-9 exhibits antibacterial activity against Gram positive *Staphylococcus aureus* (SA) but not against Gram negative *Klebsiella pneumonia* (KP). Upon its binding to SA, sB2M-9 induces clumps, a phenomenon not observed with B2M. Migration of THP-1 monocytes exposed to SA clumps was significantly greater than that to SA without clumps. sB2M-9 binds to SA, more likely as a chemokine, to facilitate THP-1 migration. As a whole, we demonstrated that REC release a novel chemokine with antibacterial activity that is shed from B2M to facilitate THP-1 migration.

The innate immune system, serving as the first line of defense, provides a non-specific protection against a large number of pathogens. Toll-like receptors (TLRs) mediate a large array of innate immune responses to pathogen components, named pathogen-associated molecular patterns (PAMPs)^1^. Their activation leads to initiate MAPK- and NF-kB-dependent cascades that culminate in a proinflammatory response^2^. This process involves the inducible expression of a large array of genes of the innate immune responses^3^,^4^ such as cytokines^5^,^6^, chemokines^7^, adhesion molecules^8^ and a broad-spectrum of antimicrobial substances, e.g. defensins^9^. Alternatively, innate immune responses can be activated through TLR-independent pathway such as the interleukin-1 receptor (IL-1R) which shares considerable homology with TLRs in the cytoplasmic region, called Toll-IL-1R domain^10^,^11^.

Inflammation, considered as a mechanism of the innate immunity, comprises a series of cellular responses including tissue turnover. By doing this, the process of inflammation triggers the degradation of extracellular matrix (ECM) by matrix metalloproteinases (MMPs). Indeed, MMPs display functions other than degradation of the ECM. MMPs were shown to participate to a range of processes, such as ligand shedding^12^, activation of cytokines^13^, antimicrobial peptides (AMPs)^14^,^15^ and chemokines^16^,^17^,^18^, often potentiating the activities of these proteins.

AMPs are usually derived from larger precursors that contain signal peptides. Some other AMPs are produced from larger proteins cleaved by proteolysis, such as buforin II^19^, lactoferricin^20^ and LL-37^21^. With respect to the negative charge conserved on the membrane surface of pathogens, almost all AMPs consist of cationic and hydrophobic domains at physiological pH^22^. Based on this feature, cleavage and release (shedding) of membrane-associated proteins display a mechanism for critical regulatory step in many normal and pathological processes. The secretions of respiratory epithelial cells (REC) provide a natural barrier of host defense against inhaled microbes. An array of antimicrobial molecules from several families are produced, including lysozyme, lactoferrin, collectins, as well as the cationic defensins, LL-37, elafin, and secretory leukocyte protease inhibitor (SLPI), which are secreted constitutively and/or inducibly by epithelial cells^23^. Inhibition of antimicrobial activities or expressions may result in an increased susceptibility to pathogens, implying that antimicrobial products play an important role in the control of airway infections. Chemokines are chemoattractant cytokines that serve as critical mediators of cell migration, particularly in the immune system. To date, 46 chemokine ligands and 20 G protein-coupled chemokine receptors have been described^24^. Chemokines orchestrate a wide range of physiological and pathological processes, including immune surveillance^25^, modulation of effector cell function within tissue^26^, inflammation^27^, tumorigenesis and cancer metastasis^28^. Of note, some chemokines display chemotaxis in part, and also act as AMPs that are so called AMP chemokines^29^. For example, chemokine CXCL14 is a broad-spectrum AMP against respiratory tract bacteria and contributes to clearance of *Streptococcus pneumonia* pulmonary infection^30^. Similar to AMPs, many chemokines are positively charged at physiological pH while some AMPs share chemotactic activities with chemokines as well^31^.

In the post genomic era, proteomic approaches allow the simultaneous analysis of a wide array of proteins contributing to our understanding of protein/peptide functions by differential display and quantitative measurement of them in cells. So, the present study was based on the use of cationic chip with surface enhanced laser desorption ionization-time of flight (SELDI-TOF) mass spectrometry (MS) to analyze the expression of cationic peptides in response to IL-1β challenge of human REC. A panel of low molecular secreted molecules was found upon their challenge with IL-1β. We focused on one of these molecules, a cleaved form of β2-microglobulin (B2M), to address primary structure of amino acids sequence and biological functions.

## Results

### Small cationic proteins secreted by REC upon IL-1β activation

**Figure 1:**
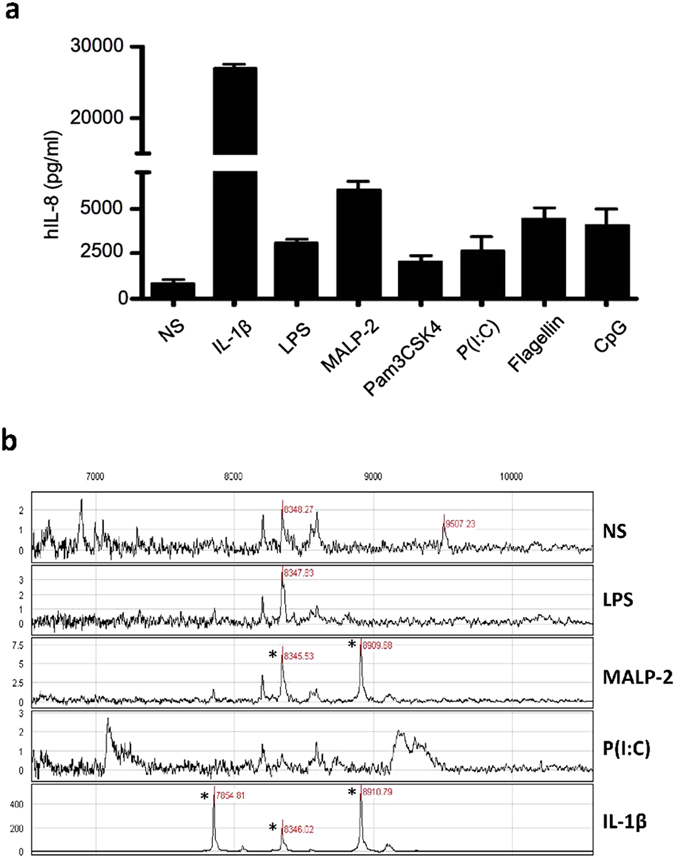
IL-8 secretion and SELDI-TOF-MS spectrum of cationic peptides present in the supernatants of REC activated by different PAMPs and IL-1β. (**a**) FCS-free media were collected from 24 h culture of A549 in response to non-stimulation (NS), IL-1β (1 ng/ml), LPS (1 μg/ml), MALP-2 (60 ng/ml), Pam3CSK4 (100 ng/ml), P(I:C) (5 μg/ml), flagellin (50 μg/ml) and CpG ODN (100 ng/ml). IL-8 production in cell supernatants was measured by ELISA. Data are means ± S.D. of three separate experiments with values expressed as in pg/ml. (**b**) Comparison of A549 responses to LPS (1 μg/ml), MALP-2 (60 ng/ml), P (I:C) (5 μg/ml), IL-1β (1 ng/ml) with non-stimulation (NS). Peptides that are up-regulated after stimulation are highlighted (*) and their masses are indicated. This spectrum represents one of three analyzed samples.

Supernatants were harvested 24 h after stimulation or not of A549 by IL-1β or different PAMPs (Fig. 1). IL-8 secretion was measured to evaluate the efficiency of the treatments. It shows that REC gave a more intense response to IL-1β than PAMPs (Fig. 1a). IL-1β-stimulated samples were then analyzed by using Protein Chip system (Ciphergen Biosystem, Freemont, CA) based on the integration of chemically modified array with SELDI-TOF MS analysis. Four different protein arrays named CM10, Q10, H4 and IMAC were initially used to bind molecules present in the supernatants of A549. CM10 gave the best signal intensity. By screening on CM10 array, the molecular masses between 1000~30,000 Da were surveyed and a cluster of small proteins, with molecular weight of 7854, 8346 and 8910 Da, was found in the supernatant of activated A549 compared to resting A549 (Fig. 1b). Of note, there was no significant signal found beyond 10 kDa.

### Identification of the protein cluster as NAP3, CXCL5 and a 9 kDa fragment of B2M

**Figure 2:**
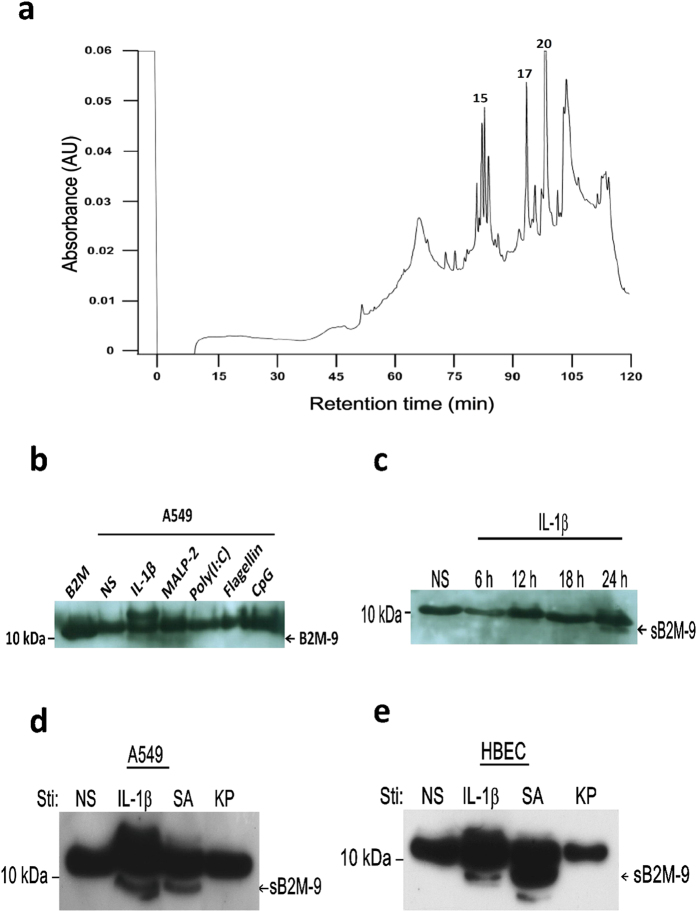
Separation and identification of sB2M-9 in the supernatant of REC upon IL-1β and bacterial stimulation. (**a**) 50 ml of IL-1β-stimulated A549 culture medium for 24 h was incubated with 200 μl of cationic exchange (CM) beads. After CM extraction, the fraction was separated by RP- C18 HPLC as described in Methods section and the elution was monitored at 214 nm. The peaks containing the equivalent masses as those determined by CM10/SELDI-TOF are marked. Peak 15, 17 and 20 correspond to mass 7854, 8910, and 8345, respectively. (**b**) FCS-free media were collected from 24 h culture of A549 in response to non-stimulation (NS), IL-1β (1 ng/ml), MALP-2 (60 ng/ml), P(I:C) (5 μg/ml), Flagellin (50 μg/ml) and CpG ODN (100 ng/ml). Western blotting of the cationic extracts of culture medium was performed by using anti-B2M polyclonal antibodies, where B2M as a control. (**c**) Time course of sB2M-9 production was determined in A549 upon IL-1β (1 ng/ml) stimulation as compared with non-stimulation (NS). The cationic extracts of culture medium were analyzed by western blotting. (**d**,**e**) sB2M-9 was detected in the secretion of (**d**) A549 and (**e**) HBEC upon IL-1β (1 ng/ml), SA (1.2 × 10^4^ cfu/ml), and KP (1.2 × 10^4^ cfu/ml) treatment.

In order to identify the interesting cluster of protein peaks found on CM10/SELDI-TOF spectrum, another batch with lager quantity of IL-1β-stimulated A549 culture medium was prepared for HPLC purification and followed by N-terminal microsequencing analysis. Firstly, the secreted molecules from REC were enriched by CM beads extraction, which have the same property as CM10 array for the binding of cationic proteins. The eluted fraction from CM beads was then subjected to RP-C18 HPLC purification. More than 20 peaks were obtained (Fig. 2a). To know which fractions of HPLC profile corresponded to the previous masses found on CM10/SELDI-TOF, the mass of each individual peak was determined by using a golden chip/SELDI-TOF MS. Among them, peak 15, 17, and 20 containing the equivalent masses as those determined by CM10 protein chip with SELDI-TOF were collected and their N-terminal amino acid sequences were determined by automatic sequencing using Edman degradation. By searching through protein database (NCBI: http://www.ncbi.nih.gov and Swiss-Prot: www.ebi.ac.uk/swissport), it reveals that peak 15, 17 and 20 match precisely with the molecular N-terminal sequences of NAP3, B2M, and CXCL5, respectively (Supplementary Table S1). The mature form of B2M with a molecular mass of 11729 Da did not fit at all with the detected present mass of 8910 Da. Therefore, a cleaved form of 9 kDa B2M derived from the mature form of B2M was suspected to be produced by REC upon IL-1β activation. We thus focused our mind on this molecule, that we named it hereafter shed 9 kDa B2M (sB2M-9). In order to further confirm the production of sB2M-9 in response to IL-1β and different PAMPs treatment of A549, the supernatant was analyzed by western blotting and showed an approximate 9 kDa band revealed by B2M antibodies (Fig. 2b). These data support that sB2M-9 is a moiety shed from native B2M. In agreement with the SELDI-TOF data (Fig. 1b), MALP-2 also induced the production of sB2M-9. Of note, time course study of sB2M-9 secretion indicates that it is present in the supernatant of A549 upon a 24 h IL-1β treatment (Fig. 2c). In addition, sB2M-9 production was found in both A549 (Fig. 2d) and HBEC (Fig. 2e) upon SA but not KP stimulation. Together, these data support that stimulation with MALP-2, a component of Gram positive bacteria, but not LPS, a component of Gram negative bacteria, could lead to sB2M-9 production in REC (Fig. 1b).

### Elucidation of amino acid sequence of sB2M-9

**Figure 3:**
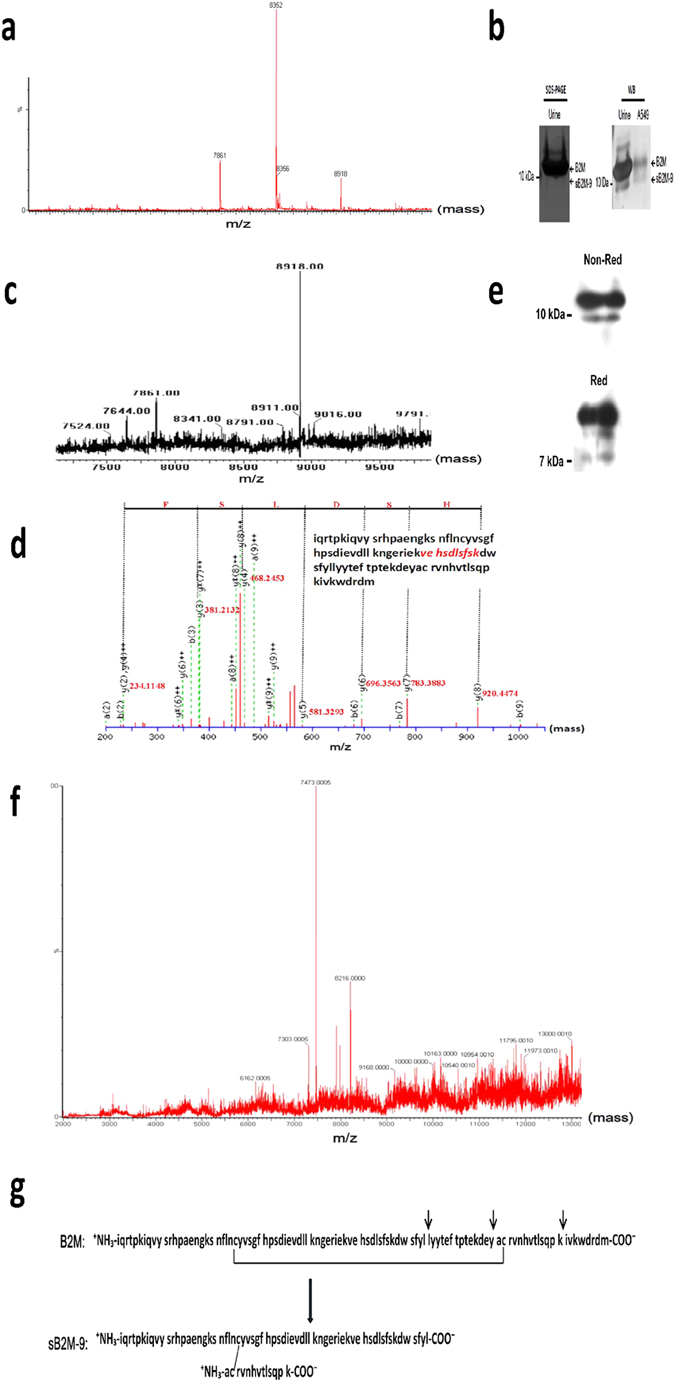
Determination of the primary sequence of sB2M-9. (**a**) After desalting, CM extract of IL-1β-stimulated A549 culture medium was analyzed by ESI-MS as described. (**b**) 5 μg of commercial native B2M of human urine origin was resolved by a 16.5% non-reducing SDS-PAGE with silver staining (left). An aliquot (0.5 μg) of commercial native B2M was analyzed by western blotting as compared with the cationic extract of A549 culture medium (right). (**c**,**d**) Mass spectra of commercial native B2M, prior extracted by binding of CM beads at pH 6.5, were shown by (**c**) ESI-MS analysis and by (**d**) ESI-MS/MS, in which a y-ion series was shown and the sequence matched with B2M was written in Italic letter code. (**e**) The binding fraction at pH 4.0 in (**a**) was reduced (Red) with dithiothreitol as compared with non-reduced (Non-Red) form and analyzed by western blotting. (**f**) Mass spectrum of the reduced sample in (**c**) was shown by ESI-MS. (**g**) The proposed amino acid sequence of sB2M-9 is derived from mature B2M, on which three expected cleavage sites are shown by short arrow head.

As molecular masses determined by SELDI-TOF did not precisely reflect the true masses of NAP3, B2M and CXCL5, the sample was further analyzed by C4-ESI-MS. It shows that the mass spectrum from ESI-MS was a little bit different from the SELDI-TOF pattern (Fig. 3a). In fact, the measured masses of 7861 Da and 8352 Da for NAP-3 and CXCL5 respectively, fit well with the theoretical masses of 7861.2 Da and of 8352.87 Da, respectively. The very good fitting of these masses serve as two reference molecules and indicates that the molecular mass for sB2M-9 is precisely 8918 Da instead of 8910 Da given by SELDI-TOF analysis. Then, the primary structure of sB2M-9 was elucidated by several experimental approaches. Firstly and as described above, the Edman degradation of N-terminal sequence showed that sB2M-9 starts with the beginning of mature B2M, namely iqrtpkiqv (Supplementary Table S1). sB2M-9 was further recognized by specific antibodies (N-19) against the N-terminus of mature B2M by western blotting (Supplementary Fig. S1). Secondly, in a survey of commercial native B2M of human urine origin by non-reducing tricine SDS-PAGE with silver staining we identified a tiny band of approximate 9 kDa (Fig. 3b, left) which was equivalent to sB2M-9 (Fig. 3b, right). This 9 kDa B2M corresponded to a 8918 Da molecule as further demonstrated by binding of CM beads at pH 6.5, followed by ESI-MS analysis (Fig. 3c) and its analysis by ESI-MS/MS, revealed a partially matched sequence with the mature B2M at V^49th^ ~K^58th^ (Fig. 3d). Thirdly, to figure out whether sB2M-9 is a continuous or discontinuous structure, supernatant of IL-1β-treated REC was resolved on a reducing tricine SDS-PAGE with immunoblotting. It shows that reduction of the extract could produces an approximate 7 kDa fragment of B2M as compared with the non-reducing status, indicating sB2M-9 is composed of a discontinuous two-chain cross-linked by a disulfide bond (Fig. 3e). In parallel, a reducing process applied to the 9 kDa moiety of commercial B2M generates a molecule of 7473 Da, referring to the partial sequence of mature B2M at I^1st^~L^64th^ (the theoretical mass, 7471.35 Da) (Fig. 3f). Accordingly, the amino acid sequence of sB2M-9 consists of 77 residues which is composed of a discontinuous two-chain, containing a long chain: I^1st^~L^64th^ and a short chain: A^79th^~K^91st^ of mature B2M, based on free software calculation (http://au.expasy.org/tools/pi_tool.html) (Fig. 3g). This result reveals that sB2M-9 is a fragment of mature B2M, on which three putative cleavage sites are shown.

### Identification of sB2M-9 in clinical samples

**Figure 4:**
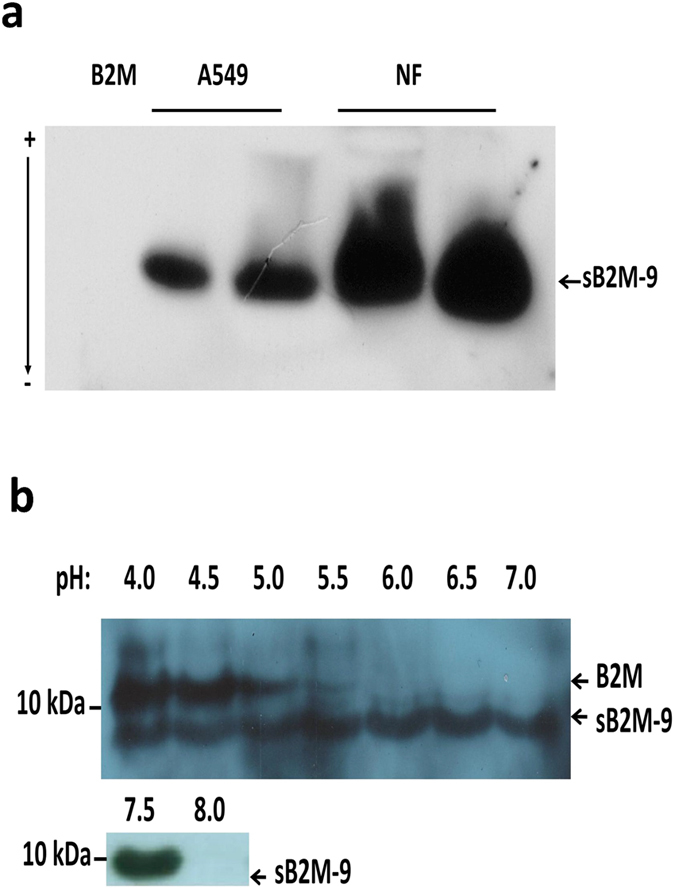
pI titration of sB2M-9. (**a**) Processing of NF extract was described in the Methods section, 10 μg of NF extract from two individuals and 5 μg of cationic extract of IL-1β-stimulated A549 culture medium was resolved on a 15% native PAGE composed of stacking gel with 1 M KOH/acetic acid buffer, pH 6.8. Positively charged proteins migrate from anode (+) toward cathode (−) were shown, followed by western blotting. Native B2M (0.5 μg) carried negative charge could not move into gel as a negative control. (**b**) 3 ml of IL-1β-stimulated A549 culture medium was adjusted to the indicated pH by 0.5 unit interval and bound to CM beads. After elution and desalting, an aliquot of samples was resolved on non-reduced tricine SDS-PAGE, and analyzed by immunoblotting.

To know whether sB2M-9 is present in human nasal airway, nasal fluid (NF) from two healthy individuals was homogenized and extracted with 0.5% acetic acid, and resolved by native polyacrylamide gel electrophoresis (PAGE), in which the stacking gel was made with 1 M KOH/acetic acid buffer, pH 6.8. Proteins with their pI higher than the pH (*i.e*. 6.8) become positively charged and therefore migrate from anode toward cathode. Figure 4a shows a cationic molecule in NF migrating to the same distance as sB2M-9 from A549 by western blot analysis. However, native B2M (pI = 6.02), a negative control, carried negative charge at pH 6.8 and could not migrate into stacking gel. Thus, it implies that the same molecule with pI higher than 6.8 is present in the culture medium of REC and clinical samples.

### Titration of the isoelectric point (pI) for sB2M-9

Proteins or peptides with a pI higher than the pH of binding condition bind onto cationic beads and begin to dissociate from the beads at about 0.5 pH unit from their pI^32^. Based on this concept, a series of titrations were made by binding supernatant of IL-1β-treated REC to CM beads. As compared with the native B2M which binds only at pH below 5.5, the binding ability for sB2M-9 to CM beads can be maintained up to pH 7.5 (Fig. 4b). In addition, the binding fraction of the supernatant at pH 7.5 containing a molecule of 8918 Da was also demonstrated by ESI-MS. It implies that pI of sB2M-9 should be as high as 8.0. This fit well with the pI of proposed structure of sB2M-9 according to free software calculation (http://au.expasy.org/tools/pi_tool.html). This feature supports and explains the increased pI for sB2M-9 produced by a shedding process of native B2M in REC upon IL-1β treatment.

### Biological activities of sB2M-9

**Figure 5:**
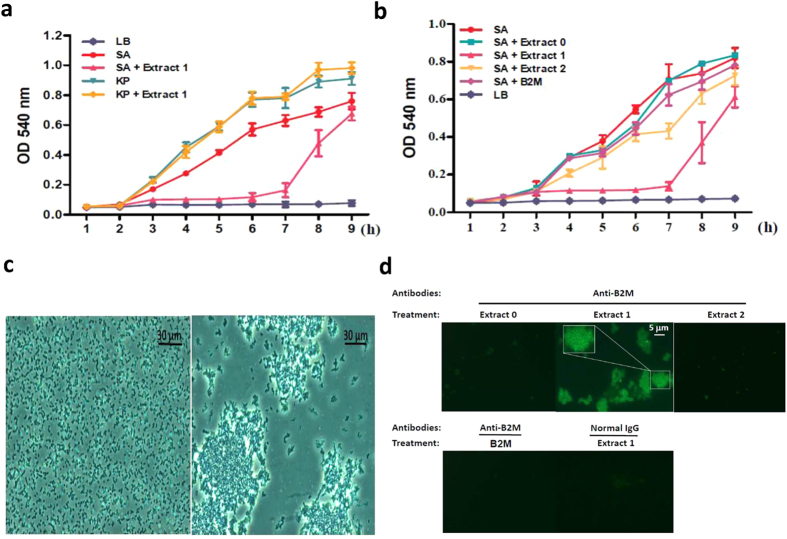
Highlighting of sB2M-9 biological properties. SA and KP were cultured at a concentration of 1.2 × 10^5^ cfu/0.1 ml in 96-well microtiter plates. (**a**) Growth of SA and KP was measured in presence of the extract of IL-1β-stimulated A549 culture medium (extract 1, 100 μg) by ELISA reader. (**b**) SA growth was measured in presence of the same volume of extracts obtained from A549 with no stimulation (NS, as extract 0), stimulated by IL-1β (extract 1) and stimulated by IL-1β with B2M depletion (extract 2), respectively. B2M depletion of extract 1 was done by immunoprecipitation using protein A/G plus agarose (Santa Cruz Biotechnology) conjugated with polyclonal anti-B2M antibodies. SA growth supplemented with 0.5 μg of commercial B2M alone was also performed. (**c**) 5 μl of SA culture broth at 5 h was observed under a phase contrast microscope (×400) (left), as compared with that co-cultured with extract 1 (right). (**d**) Clumping bacteria were collected at 5 h (**a–c**) and immunostained with anti-B2M polyclonal antibodies, followed by secondary antibodies conjugated with FITC shown under fluorescent microscopy (×400), where zoomed image (upper) corresponding to boxed areas.

CM extract of culture medium from A549 was tested for its antimicrobial activity by a bacterial inhibitory assay. In a kinetic study of bacterial growth over 9 h, the extract of supernatant of IL-1β-stimulated A549 (extract 1) displays antibacterial activity against SA, but not KP (Fig. 5a). Nevertheless, this effect could last for only a few hours. The increased growth of SA after 7 h indicates that there is a temporary inhibition instead of truly killing bacteria (Fig. 5a,b). The inhibitory effect on SA growth was diminished, although not totally suppressed, by the depletion of B2M from extract 1 (as well for extract 2) using immunoprecipitation, indicating that B2M may play a role of antimicrobial molecule against SA (Fig. 5b). However, native B2M alone exhibited very limited inhibition to SA growth. Of note, it is evident that extract 1 (Fig. 5c, right) as compared with extract 0 (Fig. 5c, left), inhibited bacterial growth by formation of SA clumps. However, extract 2 or native B2M alone could not display the same effect as extract 1 on the bacteria. This phenomenon, in agreement with bacterial growing curve (Fig. 5a,b), was observed as early as the titer of SA going up at 3 h and it disappeared after 7 h. This result implies that a molecule derived from B2M (*i.e*. sB2M-9), rather than native B2M, displays antibacterial activity to restrict SA growth during the challenge. In order to explore the role of sB2M-9 on SA clumping, SA co-cultured with extract 0, 1 and −2 for 5 h, were respectively collected and observed by using immunofluorescent staining. It clearly shows that B2M-derived molecules in extract 1 could bind the surface of SA clumps (Fig. 5d, upper panel). This phenomenon was neither observed by treatment of extract 0 or extract 2, nor by native B2M alone. Here, we rule out the immunostaining from cross reaction by normal IgG (Fig. 5d, lower panel). Together, these data support that sB2M-9 with higher pI value binds the surface of SA and is responsible for antibacterial potential of REC.

### Effect of sB2M-9 on THP-1 migration

**Figure 6:**
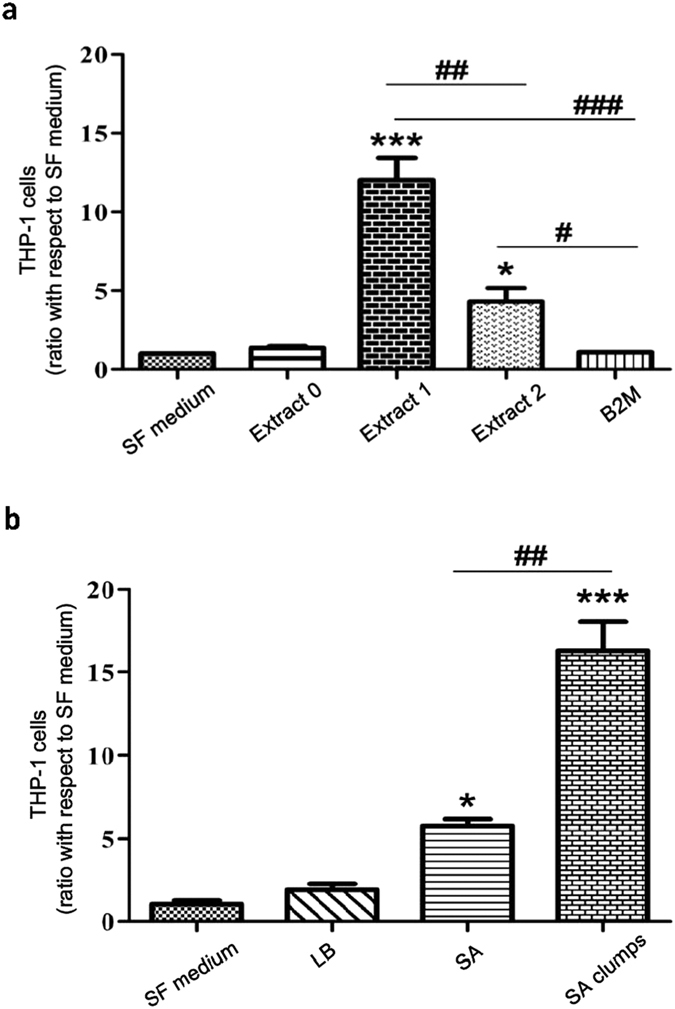
THP-1 monocytes migration in response to sB2M-9. THP-1 cells at a density of 1 × 10^4^ cells/ml were seeded in 24-well Transwell. Cationic extract of A549 secretion, bacterial inhibition assay and migration assay were described in Experimental Procedures section. (**a**) Migration of THP-1 cells in response to extract 0, extract 1 and extract 2 was compared with that to serum free (SF) medium. B2M (0.5 μg) alone was used as negative control. (**b**) SA (1.2 × 10^5^ cfu/0.1 ml) were cultured alone in 96-well plate or co-cultured with 100 μg of extract 1 for 5 h until the formation of clumping bacteria. SA and SA clumps were harvested and washed with LB. Then numbers of migrated cells in response to SF medium, LB, SA and SA clumps were counted. Numbers of migrated cells were normalized against that for SF medium to produce relative ratio, mean ± SEM, n = 4. *P < 0.05, ***P < 0.001 vs. SF medium; ^#^*P* < 0.05, ^##^*P* < 0.01, ^###^*P* < 0.001.

Given that sB2M-9 could be also detected in the culture medium of SA-infected A549, we suspected, as a result of sB2M-9 binding on the surface of SA clumps, that monocytes might be recruited to the SA clumps. To determine whether the presence of sB2M-9 is correlated with monocyte recruitment, chemotaxis assay was performed to compare the effect of extract 1 with extract 2 on THP-1 migration. After a 24 h seeding, migration of THP-1 exposed to extract 1 was significantly greater than that exposed to extract 2. However, THP-1 cells remain as the control group when they were exposed to an equivalent amount of native B2M (0.5 μg) alone (Fig. 6a). Furthermore, in order to confirm that sB2M-9, other than native B2M, binds SA clumps and facilitates monocyte migration, THP-1 cells were exposed to SA compared with that to SA clumps. Here, SA and SA clumps were harvested at 5 h of bacterial growth in presence of extract 1 as shown in Fig. 5a. Surprisingly, it clearly shows that migration of THP-1 exposed to SA clumps was significantly greater than that of cells to SA without formation of the clumps (Fig. 6b). These results reveal that sB2M-9 bound to SA is important to facilitate THP-1 migration. It also implies that sB2M-9 might display a chemokine property derived from native B2M to facilitate THP-1 migration.

## Discussion

B2M, a low molecular mass protein (*Mr* of 11729), is found as a free form in the serum and as a form noncovalently associated with the alpha chain of class I major histocompatibility complex (MHC) molecule on the cell surface^33^. Since B2M displays physiological function by maintaining structure of MHC class I, therefore, it has long been regarded as a house keeping gene^34^.

In the search of sB2M-9 produced by REC upon different PAMP stimulation, we found that REC gave more intense response to MALP-2 (a component of Gram positive bacteria) based on cationic extraction, but remained silent to LPS (a component of Gram negative bacteria) (Fig. 1b). In a parallel, REC co-cultured with SA (a Gram positive strain) instead of with Gram negative bacteria such as KP or *E. coli*, secret sB2M-9 in the culture medium. The extract of REC culture medium, therefore, exhibits antibacterial activity against SA but not KP (Fig. 5).

In the literature, we found that modified B2M had been shown in the serum of patients suffering of small cell lung cancer^35^, AIDS, chronic haemodialysis^36^, the synovial fluids of patients with rheumatoid arthritis^37^ and the urine of patient with acute tubular injury of renal allografts^38^ as well as healthy individuals^39^, suggesting a proteolytic process involved the cleavages of native B2M. It is well recognized that many of biological peptides/proteins are produced by the proteolysis from their precursor, and herein we reveal a similar mechanism in that the features of sB2M-9, including total charges and biological function, are different from its precursor B2M. Thus the increased pI value for sB2M-9 after shedding is in agreement with its antimicrobial acquired activity expressed by cationic peptides, attributing to the peptide a binding property with microbial plasma membrane that displays more negative charges. In the kinetic study of bacterial growth, we found that extract 1 displayed antibacterial activity against SA. The inhibitory effect on SA growth was significantly suppressed by the depletion of B2M from the extract. Although native B2M displaying antimicrobial property had been reported^40^, however, our own assay for native B2M using either kinetic study (Fig. 5b) or radial diffusion assay could not support such viewpoint. It implies that B2M displays antibacterial activity possibly at the post translational level through formation of sB2M-9. Previously, a putative antimicrobial activity for B2M was also demonstrated in human airway fluid^41^. In that, a B2M molecule of approximate 7–10 kDa (instead of 11.7 kDa for B2M) displaying antibacterial activity was demonstrated. We assume that it is a cleaved B2M (*i.e*. sB2M-9) rather than the native B2M that confers the antimicrobial activity. Furthermore, a role for B2M in the innate defense was characterized in B2M knockout mice to show significantly increased mortality upon KP intravenous inoculation^42^. This report clearly indicates the existence of a B2M-dependent but CD8 T-cell- and iNK T-cell-independent mechanism critical for survival during bacteremia. We speculate that an equivalent molecule to sB2M-9 shed from B2M might be responsible for cell survival during bacteremia in mice.

Interestingly, when we examined culture medium of SA co-cultured with extract 1 before 7 h, SA growth was restricted by formation of bacterial clumps as compared to that with extract 2. This effect could last for a few hours before an increased growth after 7 h (Fig. 5a,b). It implies that the extract caused temporary inhibition instead of truly bacteria killing. Neither extract 2 nor native B2M alone could trigger the formation of SA clumps in the bacterial inhibitory assay. It suggests that sB2M-9 rather than native B2M plays a role in the restriction of SA growth and the formation of SA clumps (Fig. 5c). This observation was further confirmed by fluorescent staining that shows a B2M-derived binding of the surface of SA clumps (Fig. 5d). However, native B2M alone did not exhibit the same staining as that of the extract 1. It reveals that sB2M-9, but not native B2M, binds the surface of SA clumps probably through the property of increased pI of the cleaved structure.

**Figure 7:**
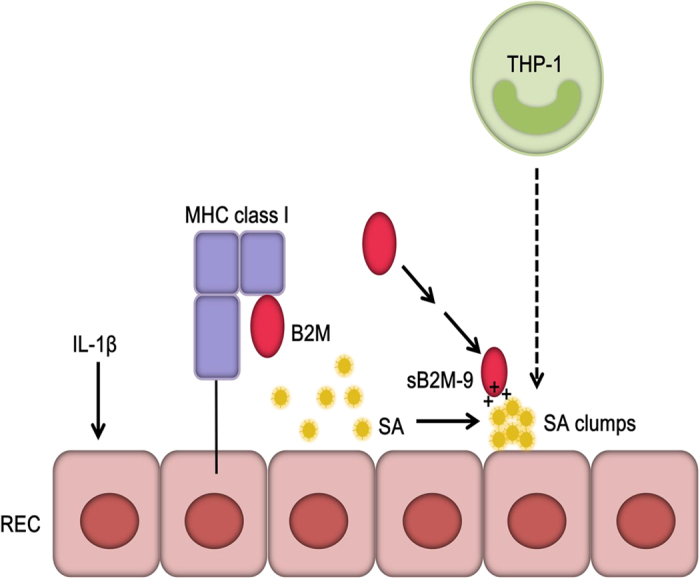
Diagram for sB2M-9 production that binds surface of SA clumps to facilitate THP-1 migration. In response to IL-1β or SA stimulation, REC release unknown proteases that cleave on B2M into sB2M-9. sB2M-9 with cationic domain inhibits SA growth by binding the surface of SA and forming clumps. Subsequently, sB2M-9-bound SA clumps trigger to THP-1 m (dashed arrow head).

It is known that monocytes move fastly towards the infected tissue and differentiate into macrophages and dendritic cells to induce an immune response^43^. Knowing the structural homology between B2M and Fc domain of IgG^44^ as well as Fc receptor expressed on monocytes^45^, the feature of sB2M-9 bound to the surface of SA clumps was therefore hypothesized to elicit migration of THP-1. Indeed, it was previously reported that B2M binds to complement and is cytophilic to macrophages as well as other cells^46^. Our results show that sB2M-9 but not native B2M enhances THP-1 migration. Furthermore, they reveal that THP-1 migration is dramatically increased by sB2M-9-bound SA clumps as compared to that by SA without forming clumps. As a cross-talk between pathogen recognizing TLRs and immunoglobulin Fc receptors in immunity has been proposed^47^, our findings suggest an existence of ligand-receptor interaction between sB2M-9-bound SA clumps and Fc receptors of THP-1 cells. These results have been consolidated into a diagram that points out the findings of this study (Fig. 7).

According to our knowledge, this is a novel identification of a cleaved B2M with antibacterial and chemotactic activity which is generated probably by distinct proteolytic cleavages. Along with other antimicrobial peptides, this would constitute an important innate defense system played by REC against lung infection.

## Methods

### Reagents and antibodies

F-12K nutrient mixture (Kaighn’s modification) (GIBCO-BRL, USA), antibiotics, glutamine, and trypsin-EDTA were obtained from Invitrogen. Fetal calf serum (FCS) was obtained from Hyclone (Logan, UT). B2M standard protein was obtained from Sigma-Aldrich Co. (St. Louis, MO). Interleukin (IL)-1β was obtained from Peprotech Asia (Israel). For immunoblotting, anti-B2M antibodies included the goat anti-human B2M polyclonal antibodies (N-19), against the N-terminal region of human B2M and the full length of human B2M (FL-119, Santa Cruz Biotechnology, Santa Cruz, CA), and horseradish peroxidase-conjugated anti-rabbit and anti-goat IgG (secondary antibody) were obtained from Thermo Fisher Scientific (Rockford, IL).

### Cell culture

A549 (ATCC, CCL-185), a type II alveolar epithelial cell line from human adenocarcinoma, and human bronchial epithelial cells (HBEC)(ATCC, CRL-3245) were maintained in 5% CO_2_ in F-12K Nutrient Mixture medium supplemented with 10% (vol/vol) FCS, 0.3 mg/ml L-glutamine, 100 U/ml penicillin, 100 μg/ml streptomycin, 0.25 μg/ml fungizone and 25 mM HEPES. These REC grown in 10 cm dishes to 90% confluence were washed and changed to serum-free of above medium, followed by stimulation with IL-1β for 24 h. THP-1 cells (ATCC, TIB-202), a human monocytic cell line, was cultured in suspension of RPMI 1640 (GIBCO-BRL, USA), 10% FCS, 2 mM L-Glutamine and 0.05 mM 2-mercaptoethanol (GIBCO-BRL, USA).

### Measurement of interleukin-8 (IL-8)

REC were seeded at 1 × 10^6^ cells/ml in 12-well plates (Costar, Corning, NY, USA) until 90% confluence, and then changed to FCS-free medium after one washing of the cell monolayers. FCS-free medium was recovered after 24 h of incubation under cell culture condition. The measurement of IL-8 concentration was determined by using ELISA (R&D System, Minneapolis, MN).

### Collection and processing of nasal fluid

The methods were carried out in accordance with the approved guidelines and protocols for clinical experiment were approved by Kaohsiung Medical University Hospital Institutional Review Board (KMUH-IRB-20120032). The written informed consent was obtained from all subjects. Human nasal fluid (NF) was collected from healthy volunteer donors. Approximately 1 ml of NF was homogenized by using an ultra-sound homogenizer (dr. Heilscher/GmbH) and extracted by 0.5% acetic acid. The supernatant was kept in aliquots and lyophilized.

### Surface-enhanced laser desorption/ionization time-of-flight (SELDI-TOF) analysis

SELDI-TOF (Protein Chip System, Series 4000, Ciphergen, Freemont, CA) was performed to detect peptides of interest. CM10 Protein Chip Array (Ciphergen) was used to screen the culture medium of REC upon IL-1β activations. The steps for preparing CM10 chip are as follows: activation of spot surface with 100 mM Sodium Acetate (pH 4.0) for 10 min. After removing the solution, five microliters of sample were applied to the chip containing another 50 μl of 100 mM Sodium Acetate (pH4.0)/0.1% TritonX-100 and incubated for 60 min at room temperature (RT). Spots were washed one time with 200 μl of 100 mM Sodium Acetate, pH 4.0/0.1% Triton X-100 for 5 min at RT and two times (200 μl) of 100 mM Sodium Acetate, pH 4.0 for 5 min at RT and two times rapid washing with milli-Q water. Chips were dried in air-suction box for 10 min. Two times of 0.7 μl of Sinapinic Acid (SPA) was applied to each spot and air-dried naturally. Chips were read by SELDI-TOF instrument with 1200 nJ laser intensity.

### Purification of IL-1β-induced peptide release

Supernatant of IL-1β-stimulated REC was adjusted to contain Sodium Acetate (100 mM, pH 4.0 in the final), and extracted with CM Ceramic HyperD F ion-exchangers (BioSepra, S.A., France). The fraction containing fragment of B2M (corresponding to mass = 8910 Da) was eluted with 100 mM Tris-HCl, pH 10.0/0.5 M NaCl and concentrated by using Speed Vac Concentrator (Savant Instrument Inc). The concentrated fraction was applied to RP-C18 HPLC column (150 × 1.5 mm, Uptishere 3 ODB, Interchim, France) and eluted with a gradient of increasing concentrations of acetronitrile (2–80%) containing 0.1% (v/v) tri-fluoroacetic acid (TFA).

### NH_2_-terminal amino acid sequence analysis of IL-1β-induced peptide

Homogeneous material from RP-C18 HPLC was subjected to chemical sequencing. Sequencing was performed on an Applied Biosystems (ABI 494 liquid phase sequencer), with on line HPLC analysis of the phenylthiohydantoin derivatives.

### Western blotting

For the study of different forms of B2M, protein samples were fractioned by electrophoresis on a 16.5% Tricine- SDS-PAGE (BioRad, USA) and transferred onto Immobilon-PSQ PVDF membrane (Millipore, Bedford, MA, USA) by using semi-dry apparatus (Hoeffer Scientific Instruments, CA). Peptides were cross-linked on membrane with 0.5% glutaraldehyde for 30 min at RT and non-specific binding sites were blocked with 5% non-fat milk in TBST for 2 h. The membrane was incubated with rabbit anti-B2M (FL119) or goat anti-B2M (N-19) polyclonal antibody (1:200) (Santa Cruz Biotechnology, Santa Cruz, CA) for overnight followed by a secondary antibody conjugated with horseradish peroxidase (1:15,000) for another 1 h. Detection was performed using the Chemiluminescent HRP Substrate (Millipore, Billerica, USA).

### Titration of isoelectric points (pI)

A series of supernatants from IL-1β-stimulated REC was titrated by binding to CM beads in the presence of 0.1 M sodium acetate at pH 4.0–9.0 for 2 h at 4° C. After washing step, the elutent by 0.1 M Tris-HCl, pH 10.0/0.5 M NaCl was desalted using Sep-Park C18 cartridges (Waters) and analyzed by western blotting as described above.

### Mass spectrometric analysis

Molecular masses of parent or shed B2M were identified by Micromass quadruple time-of-flight (ESI-Q-TOF) Global Ultima mass spectrometer equipped with a nanospray source (Manchester, UK) at Center for Resources, Research & Development of Kaohsiung Medical University. Briefly, polypeptides were separated and concentrated by capillary LC system (Water, Milford, USA) using double columns, a desalting column (C18 PepMap, 300 μm ID, 5 mm, LC Packings, Sunnyvale, USA) and a reverse-phase column (Symmetry C18, 75 μm ID, 100 mm, Waters). Peptides eluted from capillary columns were directed into the nanospray needle by a 20 μm i.d. and 90 μm o.d. fused-silica capillary. A voltage of 3.2 kV was applied to the nanosource. The mass spectrometer was operated in positive ion mode and TOF analyzer was set in the V-mode. MS/MS spectra were obtained in a data-dependent acquisition mode in which the two major multiple-charged (+2 and +3) peaks with the three most abundant ions were selected for collision-induced dissociation (CID). The scan range of precursor and fragment ions was m/z 400–3,000 and m/z 100–3,000, respectively. MS/MS spectra acquired for each of the parent ions was processed by MassLynx 4.0 software (Manchester, UK). Peptide sequences were identified by searching against protein database.

### Native polyacrylamide gel electrophoresis (PAGE)

Electrophoresis was carried out as described^48^. Briefly, separating gel (15%, acrylamide/bis solution, 29:1) was prepared using 1 M KOH/acetic acid buffer, pH 4.3, and stacking gel (4%, acrylamide/bis solution, 29:1) was made using 1 M KOH/acetic acid buffer, pH 6.8. Samples were acidified with separating gel buffer. Electrophoresis was employed by 35 mM β-alanine, pH 4.5, with cooling system for 3.5 hours, a constant 100 V for the first hour followed by the constant 200 V for another 2.5 hours.

### Bacterial inhibitory assay

*Staphylococcus aureus* (SA) and *Klebsiella pneumoniae* (KP) were kindly provided by Dr. R.N. Lee (Kaohsiung Medical University). Antibacterial activity was monitored by a liquid-growth inhibition assay in 96-well microtiter plates in a final volume of 100 μl containing bacteria at a concentration of 1.2 × 10^5^ colony-forming units (CFU)/ml in LB culture medium. The kinetics of bacterial inhibition was assessed by monitoring the absorbance at 540 nm with an ELISA Reader (Dynex Technologies, USA) after incubation at 37 °C for different periods of time.

### Chemotaxis assay

Human THP-1 monocytes were cultured at a density of 1 × 10^4^ cells/ml and Trypan blue was used to determine cell viability and number. Migration assay was conducted using 24-well Transwell (8-μm pore size polycarbonate membrane; CoStar, Bethesda, MD, USA) chambers as described previously^49^. Briefly, cells (1 × 10^4^) suspended in 400 μl of RPMI-1640 containing 10% FBS were placed in the upper chamber, whereas analyzed sample in 600 μl of serum-free RPMI-1640 was added to the lower wells. After 24 h of culture at 37 °C under 5% CO_2_/95% air, the cells on top side of membrane were removed using a cotton tip applicator, whereas the cells on the down side were fixed with methanol and stained with 0.5% crystal violet. Migrated cells were counted under a phase contrast microscope (×400); this value was then normalized against that for serum-free medium to produce relative ratio. Experiment was performed three times in triplicate wells of cells.

### Statistical analysis

Statistical significance was determined using GraphPad Prism 6 software. The data were expressed as the mean ± standard errors. Student *t*-tests were used for the comparison between two groups. One-way analysis of variance followed by Student *t*- tests was applied for comparison between more than three groups. A *P*-value < 0.05 was considered to be significant.

## Additional Information

**How to cite this article**: Chiou, S.-J. *et al*. A novel role for β2-microglobulin: a precursor of antibacterial chemokine in respiratory epithelial cells. *Sci. Rep*. **6**, 31035; doi: 10.1038/srep31035 (2016).

## Supplementary Material

Supplementary Information
