# Supplementary material for: A novel role for β2-microglobulin: a precursor of antibacterial chemokine in respiratory epithelial cells
Source: Sci Rep. 2016 Aug 9;6:31035. doi: 10.1038/srep31035 (PMC4977529; doi:10.1038/srep31035)
Supplement: Supplementary Information [file srep31035-s1.pdf]

Title: A novel role for β2-microglobulin: a precursor of antibacterial chemokine in respiratory epithelial cells

Authors: Shean-Jaw Chiou[1*], Chan-Chi Wang[1], Yan-Shen Tseng[1], Yen-Jung Lee[2], Shih-Chieh Chen[3], Chi-Hsien Chou[2], Lea-Yea Chuang[1], Yi-Ren Hong[1,3], Chi-Yu Lu[1,4], Chien-Chih Chiu[5], Michel Chignard[6,7*]

Supplementary Information

Supplementary Figure S1. sB2M-9 was recognized by N-19 antibodies which are against N-terminal sequence of B2M.

The cationic extract of IL-1β-stimulated A549 culture medium was analyzed by western blotting as described. sB2M-9 was detected using FL-119 Ab (against full-length of B2M, left) or N-19 Ab (right) which recognizes only N-terminal sequence of B2M.

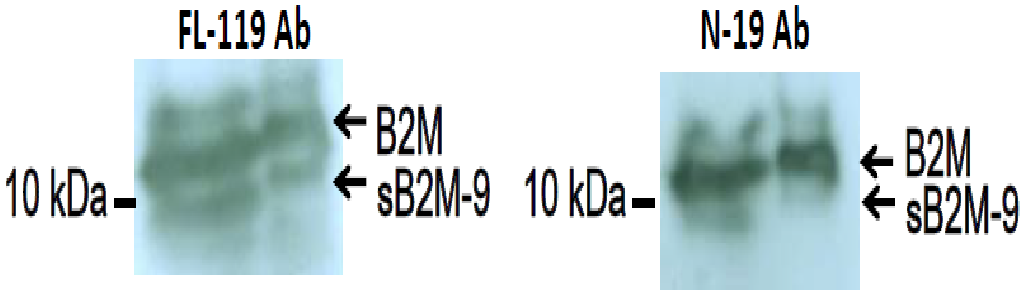

Supplementary Table S1. NH_2-terminal amino acid sequences of NAP3, B2M and CXCL5.

The N-terminal partial sequences of peak 15, 17, and 20 of HPLC profile in **Fig. 2a** were determined by Edman degradation as described in the Methods section. The single letter code for amino acids is used. The determined masses from **Fig. 1b** by SELDI-TOF and their theoretical masses are shown.

Peak	N'-sequence	Protein name	MS (Determined, Da)	MS (Theoretical, Da)
15	asvatelrcqclqtlqg	NAP-3	7854.8	7861
17	iqrtpkiqv	B2M	8910.79	11729
20	agpaaavlrelrcv	CXCL5	8346	8352.87
